# GIS ANALYSIS OF PHYSICAL ACTIVITY AND FALL RISK AMID GLOBAL WARMING: ROLES OF COMMUNITY ENVIRONMENTS

**DOI:** 10.1093/geroni/igae098.3991

**Published:** 2024-12-31

**Authors:** Yingru Li, Ladda Thiamwong, Dahee Kim, Christopher Emrich, Rui Xie, Jennifer Crook, YunYing Zhong

**Affiliations:** University of Central Florida, Orlando, Florida, United States; University of Central Florida, Orlando, Florida, United States; University of Central Florida, Orlando, Florida, United States; University of Central Florida, Orlando, Florida, United States; University of Central Florida, Orlando, Florida, United States; University of Central Florida, Orlando, Florida, United States; University of Central Florida, Orlando, Florida, United States

## Abstract

Regularly engaging in physical activity (PA) may reduce fall risk among older adults. However, hot weather amid global warming and/or less walkable community environments may negatively impact their participation in PA, especially those who live in low-income or otherwise socially vulnerable areas. This study aims to visualize spatial variations of PA and fall risk among low-income older adults (LOAs) across four communities in Orlando, Florida, during the summer months and to examine the roles of community environments on fall risk and PA. This study used data from 63 LOAs collected from May to September 2023. Self-reported fall risk was measured using the CDC STEADI fall risk checklist. PA was assessed through average active and sedentary minutes recorded by FitBit over five months. Community environments were assessed using walk scores and 31 different social vulnerability index characteristics. This study employed ArcGIS pro to visualize spatial variations and SPSS Amos to examine the roles of community environments. LOAs’ PA and fall risk varied across the four communities. Higher PA and lower fall risk were observed among LOAs in communities with higher walk scores and lower social vulnerability indexes. The results of path analysis revealed recursive relationships: higher community social vulnerability was associated with lower community walkability, which led to less active and more sedentary time among LOAs, contributing to their higher fall risk. These findings highlight the impact of community environments on LOAs’ health and well-being and suggest that actions should be taken to improve community walkability, particularly in socially vulnerable communities.

